# Barriers and Facilitators to Implementing Evidence-Based Breastfeeding Support in Miami-Dade County Maternity Wards

**DOI:** 10.1007/s10995-026-04319-3

**Published:** 2026-09-19

**Authors:** Cynthia Lebron, Vanessa Morales, Alexa Parra, Sydney Litten

**Affiliations:** 1 School of Nursing and Health Studies, University of Miami, 5030 Brunson Dr, Coral Gables, FL 33146 USA; 2 Miller School of Medicine, Department of Public Health, University of Miami, 1120 NW 14th St, Miami, FL 33136 USA

**Keywords:** Breastfeeding, Lactation consultant, Baby-friendly hospitals

## Abstract

**Objectives:**

This qualitative study assessed the breastfeeding environment across all 12 maternity hospitals in Miami-Dade County, Florida, to identify institutional factors that influence breastfeeding support.

**Methods:**

Interviews were conducted with lactation consultants and nurses and analyzed using the Consolidated Framework for Implementation Research (CFIR).

**Results:**

Hospitals varied substantially in adherence to Baby-Friendly Hospital Initiative (BFHI) guidelines, regardless of official designation. Participants identified high cesarean rates, staffing shortages, cultural misconceptions, and inconsistent clinical protocols as key barriers to breastfeeding initiation and exclusivity. Despite these challenges, many hospitals employed innovative strategies, such as virtual lactation support and community partnerships, to promote breastfeeding. Notably, lack of prenatal education and limited physician involvement were seen as critical missed opportunities.

**Conclusions for Practice:**

Results underscore the importance of coordinated, multilevel interventions that align clinical practice, staff training, and patient education. Recommendations from community partner BreastfeedMIAMI highlight the need for expanded lactation coverage, standardized staff training, culturally tailored education, and greater institutional investment. These insights can inform policy and programmatic efforts to foster more equitable, supportive breastfeeding environments in diverse hospital settings.

## Significance

Hospitals face persistent structural and cultural barriers that hinder the consistent implementation of evidence-based breastfeeding practices during maternity care. The Baby-Friendly Hospital Initiative improves breastfeeding outcomes, but full adherence varies across hospital systems due to staffing, training, and policy gaps. Qualitative findings underscore the need for coordinated, multilevel strategies that integrate clinical practice, staff training, and patient education to strengthen hospital-based breastfeeding support.

## Introduction

Breastfeeding provides an exceptional primary prevention intervention for infants and mothers alike (Binns et al., 2016; Del Ciampo & Del Ciampo, 2018). The “personalized medicine provided by human milk” (Victora et al., 2016) protects children against adverse health outcomes such as otitis media, dental malocclusions, asthma, sudden infant death syndrome, and obesity(Brahm & Valdes, 2017). Breastfeeding has positive health implications for infants that extend into adolescence and adulthood, including neurodevelopment, metabolic health, and cancer prevention (Masi & Stewart, 2024). Globally, there is a strong consensus that breastfeeding protects against preventable morbidity and mortality among infants, contributing to a reduction in sudden infant death syndrome, diarrhea, and respiratory infections (Masi & Stewart, 2024; Prentice, 2022). For nursing women, breastfeeding reduces the risk of postpartum depression (Masi & Stewart, 2024) and breast cancer, improves birth spacing, and may also reduce the risk of ovarian cancer and diabetes (Victora et al., 2016).

The World Health Organization (WHO) and the United Nations Children’s Fund (UNICEF) established the Baby-Friendly Hospital Initiative (BFHI) in 1991 as a hospital-based intervention to increase breastfeeding rates (Staff, 2013). It established guidelines for hospitals to protect and support breastfeeding using the Ten Steps to Successful Breastfeeding that must be incorporated into policy and clinical practice for a hospital to achieve BFHI status (Gomez-Pomar & Blubaugh, 2018). The Ten Steps include: (1) implementing the Critical Management Procedures to support breastfeeding; (2) ensuring staff have the knowledge, competence, and skills to support breastfeeding; (3) discussing the importance and management of breastfeeding with pregnant women and their families; (4) facilitating immediate and uninterrupted skin-to-skin contact and early initiation of breastfeeding; (5) supporting mothers to initiate and maintain breastfeeding and manage common difficulties; (6) not providing breastfed newborns any food or fluids other than breast milk unless medically indicated; (7) enabling mothers and their infants to remain together; (8) supporting mothers to recognize and respond to their infants’ feeding cues; (9) counseling mothers on the use and risks of feeding bottles, teats, and pacifiers; and (10) coordinating discharge to ensure timely access to ongoing breastfeeding support and care. Baby-friendly hospitals in the United States have elevated rates of breastfeeding initiation, duration, and exclusivity (Abrahams & Labbok, 2009; Lebron et al., 2024; Philipp et al., 2001). Moreover, there is a dose-response relationship between the number of the Ten Steps to which women are exposed and improved breastfeeding outcomes, highlighting that a Baby Friendly Hospital (which, by definition, adheres to all Ten Steps) is most beneficial for its community (Pérez-Escamilla et al., 2016).

The BFHI is widely regarded as the gold standard for hospitals committed to promoting breastfeeding initiation (Fair et al., 2021). However, BFHI designation is not mandatory, and its universal implementation may not be feasible or appropriate for all healthcare settings, given variations in institutional priorities, patient populations, and resource availability (Walsh et al., 2023). Researchers have examined barriers and facilitators to BFHI Ten Steps implementation in hospital settings in the United States and globally, identifying challenges at the patient, provider, and system levels. Commonly reported barriers include low maternal engagement with breastfeeding, high cesarean delivery rates, staffing constraints, limited staff training, provider resistance, and organizational and infrastructure limitations, alongside broader system-level factors such as policy support and leadership engagement (Kang et al., 2025; Walsh et al., 2023). Meta-analytic and systematic reviews have synthesized evidence on the effectiveness and implementation of the BFHI and related breastfeeding support interventions, while also identifying important gaps, including variability in implementation fidelity, heterogeneity in study designs, and the need for higher-quality longitudinal and experimental research, particularly to strengthen evidence across diverse healthcare and policy contexts (Habte et al., 2025; Moret-Tatay et al., 2025; Walsh et al., 2023).

In response to this evidence base, the BFHI revised its implementation guidelines to address the most persistent barriers and provide hospitals with targeted strategies for overcoming them. Despite these advances, important gaps remain. Most existing studies examine BFHI-designed facilities or implementation at the national or international level, leaving local implementation dynamics, particularly in racially and ethnically diverse, under-resourced urban settings where the majority of hospitals lack BFHI designation, understudied (Organization, 2009). Furthermore, few studies have applied structured implementation science frameworks, such as the Consolidated Framework for Implementation Research (CFIR), to systematically characterize the multilevel determinants of breastfeeding support across an entire county’s maternity ward landscape. Understanding these local determinants is critical to designing targeted, context-sensitive interventions.

In this qualitative study, we conducted interviews with hospital personnel to evaluate the breastfeeding environment in maternity wards across hospitals in Miami-Dade County. The objective of this study was to qualitatively assess the breastfeeding environment across all 12 maternity hospitals in Miami-Dade County, Florida, to identify institutional factors that influence breastfeeding support. This qualitative design was well suited to characterizing *how* and *why* breastfeeding support varies across Miami-Dade County maternity wards, capturing institutional context and provider perspectives that outcome data alone would not reveal. Interviews were guided by Baby-Friendly USA’s Ten Steps to Successful Breastfeeding, and the resulting data were analyzed using the CFIR to systematically characterize the multilevel determinants of the breastfeeding environment. By examining institutional breastfeeding support, we aim to identify factors that influence adherence and barriers to evidence-based breastfeeding practices and inform targeted interventions to optimize maternal and infant health outcomes.

## Methods

### Study Setting

This study was conducted in Miami-Dade County, Florida, a large, densely populated urban county with one of the most racially and ethnically diverse populations in the United States. According to census estimates, Miami-Dade County is home to approximately 2.7 million residents, of whom over 68% identify as Hispanic or Latino. This demographic context is relevant to breastfeeding practices because cultural beliefs, generational norms, family influence, and language barriers can shape infant decisions. Florida has one of the highest cesarean delivery rates in the nation, at 35.9%. High cesarean delivery rates have well-documented implications for breastfeeding initiation, as it is associated with delayed skin-to-skin contact, maternal sedation, mother-infant separation, and early formula supplementation, all of which are barriers to establishing exclusive breastfeeding (Li et al., 2021).

At the time of this study, 3 of the 12 maternity hospitals in Miami-Dade County held Baby-Friendly designation, all of which were part of the same nonprofit academic medical system. The remaining 9 hospitals had not achieved BFHI designation, though many incorporated BFHI-aligned practices into their protocols to varying degrees. The predominance of non-BFHI hospitals, combined with wide variation in institutional resources and breastfeeding infrastructure, provided a unique opportunity to examine implementation barriers and facilitators across the county.

### Participants and Procedures

As the goal of this study was to interview key informants from each of the 12 maternity wards in Miami-Dade County, Florida, participants included 12 individuals, each representing one of the maternity wards. Eligible participants were identified by study staff as highly knowledgeable about the breastfeeding environment in their hospitals, based on their role involving regular interaction with perinatal and postnatal women (e.g., lactation consultant, nurse educator, or clinical nurse). Participants were required to be currently employed in a maternity ward within Miami-Dade County and to hold a role with direct responsibility for or regular involvement in breastfeeding support or education.

We used purposive and snowball sampling to identify participants who met these criteria, with snowball sampling employed when direct outreach did not yield a respondent from a given hospital. This community-engaged study was born of a need identified with a community partner, BreastfeedMIAMI, a local non-profit organization that advocates, promotes, and supports breastfeeding to empower families throughout Miami-Dade County. BreastfeedMIAMI was interested in learning about the birthing landscape across the county in order to understand and potentially partner with hospitals to improve the breastfeeding environment. BreastfeedMIAMI provided initial contacts, and a study team member independently searched hospital websites to identify additional potential participants. The research team used the list to contact hospital personnel by phone and email. Each hospital contributed one participant to the study; this design was intentional, as the goal was to capture the institutional perspectives of each maternity ward. Participants who agreed to participate completed a one-hour semi-structured interview via Zoom video conference between May and June 2023. Participants were compensated $100 for their time and contributions. Following the conclusion of the analysis, a summary of the results was shared with all participants. Participants were invited to review the findings to ensure accuracy and provide feedback via email on the interpretation of the data.

All study procedures were approved by the University of Miami Institutional Review Board. The study was deemed an exempt study, as participants were acting as key informants providing information about institutional policies and resources rather than as research subjects disclosing sensitive information. All methods were performed in accordance with the relevant guidelines and regulations.

All interviews were conducted by female researchers with training in public health. To enhance the reliability of data collection, all interviews were conducted using the same standardized interview guide by the same two study team members. The full study team conducted the data analysis. It included an Assistant Professor with a PhD whose research focused on the impact of breastfeeding on maternal and child health outcomes, two doctoral students, and an undergraduate student.

### Interview Guide

The lead author developed a semi-structured interview guide, informed by Baby-Friendly USA’s *Ten Steps to Successful Breastfeeding* (See Appendix). The guide allowed the research team to assess participants’ responses against the gold standard, the *Ten Steps to Successful Breastfeeding*, as a benchmark for evaluating how closely hospital resources and practices aligned with Baby-Friendly standards. The interview guide was reviewed by BreastfeedMIAMI, our community partner organization, to confirm that the questions comprehensively and accurately captured the breastfeeding-related practices, resources, and challenges relevant to maternity wards in Miami-Dade County before data collection. Interviews were conducted virtually and were recorded and transcribed for analysis.

### Theoretical Framework and Codebook Development

To evaluate the breastfeeding environment in maternity wards across Miami-Dade County, we employed a deductive thematic analysis approach informed by an iterative, data-driven refinement of construct defintions. Deductive thematic analysis is used in qualitative research when theoretical concepts or frameworks guide the identification and analysis of themes within the data (Roberts et al., 2019). We used a contextually adapted version of the updated Consolidated Framework for Implementation Research (CFIR) as our guiding framework. The CFIR, which is commonly used in implementation science research, categorizes determinants that influence the implementation of interventions into five domains: intervention characteristics, outer setting, inner setting, characteristics of individuals, and process of implementation (Damschroder et al., 2022). We selected the CFIR because it provides a structured approach for evaluating factors across multiple settings nested within the hospital setting that may affect the successful implementation of the intervention.

For the purpose of this study, the intervention was operationalized as the breastfeeding environment within the hospital setting, which is broadly comprised of maternity wards’ design, the availability and qualifications of staff to provide breastfeeding support, the resources available to educate mothers, the support provided to new mothers, the policies related to breastfeeding, and the role of breastfeeding in discharge planning. We further operationalized established CFIR constructs to reflect the breastfeeding context. Table 1 presents the full list of codes used in analysis (organized by CFIR domain) along with their operational definitions as applied in this study. These operational definitions are provided in Table 1. We then used an inductive approach to refine the operational definitions of these established CFIR constructs, not to introduce new codes, ensuring that each definition accurately reflected how the construct was expressed in participants’ accounts of the breastfeeding environment. Specifically, the study team iteratively reviewed interview transcripts to revise and finalize the construct-level operational definitions presented in Table 1, while retaining CFIR’s existing set of domains and constructs. Specifically, the study team iteratively reviewed interview transcripts for the CFIR codes that fit the present study. Combining deductive and inductive approaches allowed for a flexible analysis while maintaining a structured framework for evaluation (Roberts et al., 2019). The full interview guide, including each question’s correspondence to the Ten Steps and its anticipated CFIR domain, is provided in Appendix A.

**Table 1:** Operationalized CFIR constructs for assessing breastfeeding environment in hospital settings

CFIR domain/construct	Operationalization
Innovation domain	
Innovation complexity	The perceived challenges or complexity of breastfeeding initiation and the delivery of breastfeeding support services and education to mothers during labor and delivery in a hospital setting.
Innovation evidence-based	Breastfeeding support services have substantial evidence that demonstrates its effectiveness.
Outer setting domain	
Policies & laws	The presence and impact of laws, regulations, professional guidelines, and accreditation standards that directly influence the implementation and delivery of breastfeeding support services in hospital settings.
External pressure	The influence of external factors, outside the direct control of implementation leaders and providers, that impact the implementation and delivery of breastfeeding support services.
Local attitudes	The sociocultural values and beliefs of the hospital support implementation of breastfeeding support services.
Critical incident	Unanticipated or major events such as COVID-19 disrupt the implementation of breastfeeding support services in the hospital setting.
Financing	There is funding from external systems such as insurance and grants that allow for the implementation of breastfeeding support services in the hospital setting.
Inner setting domain	
Access to knowledge & information	The availability and accessibility of resources, training, and guidance that enable implementation leaders and providers to effectively implement and deliver breastfeeding support services.
Work infrastructure	The organization of staffing, scheduling, and task/responsibility distribution among lactation consultants and other staff that supports their ability to deliver breastfeeding support services.
Available resources	The availability of physical, financial, and human resources that are essential for the successful implementation and delivery of breastfeeding support services.
Recipient-centeredness	There are shared values, beliefs, and norms among hospital staff regarding the importance of educating mothers about breastfeeding while also addressing their individual needs, preferences, and well-being to ensure effective support and care during their breastfeeding journey.
Materials and equipment	There are ample supplies available for the implementation and delivery of breastfeeding support services.
Tension for change	The attitude towards the situation in the hospital is that it is intolerable and must be changed. Thus, advocating for the implementation of breastfeeding support services.
Physical infrastructure	The hospital layout is designed as to support the implementation of breastfeeding. Further there are tangible materials and features that allow for the functional performance of breastfeeding support services.
Learning-centeredness	The hospital culture has shared values, beliefs, and norms that center around continual improvement and utilizing well-informed scientific data to inform breastfeeding.
Relative priority	The implementation and delivery of breastfeeding support and practices is of utmost importance in the hospital setting.
Communications	There are high quality informational sharing practices within the hospital that allow for effective and swift communication among staff members.
Funding	There is funding available in order to implement and deliver breastfeeding support services within the hospital.
Mission alignment	The implementation and delivery of breastfeeding support services aligns with the overarching purpose and goals of the hospital.
Space	There is ample physical space in order to implement and deliver breastfeeding support services.
Culture	The staff share values and norms that support the implementation and delivery of breastfeeding support services.
Human equality- centeredness	Shared values, beliefs, and norms among hospital staff regarding the equal worth of all patients, reflected in efforts to ensure equitable access to breastfeeding resources and support regardless of factors such as immigration status or socioeconomic background.
Individuals domain	
Mid-level leaders	Moderate-level leaders, such as nurse managers, nurse directors, unit directors, pediatricians, and doctors, who report to senior leadership, and supervise staff involved in breastfeeding support services.
Implementation leads	Lactation consultants who lead and direct effects to implement breastfeeding initiation and adoption.
Implementation facilitators	Nurses who assist, coach, or provide support to implement breastfeeding support services.
High-level leaders	Individuals with higher authority such as the hospital CEO or director that oversee the entire hospital.
Opinion leaders	Individuals such as family members or friends that have informal influence over the attitudes and beliefs of mothers.
Implementation process domain	
Assessing context	Collect patient information to identify and assess barriers and facilitators to the breastfeeding initiation and adoption in the hospital setting.
Assessing needs	Collect information about the priorities, preferences, and needs of mothers and families regarding breastfeeding initiation and adoption in the hospital setting.
Doing	Implementing the innovation in small, adaptable steps in order to find the best practices of delivery breastfeeding support services.
Teaming	The intentional coordination and collaboration of healthcare professionals, including implementation leaders, mid-level leaders, and implementation facilitators
Adapting	Modifying the breastfeeding support services and hospital practices in order to find the optimal version of integrating them into the hospital setting.

### Coding Procedures

Two members of the study team independently coded each transcript line-by-line using Dedoose, a qualitative data analysis software (QDAS), applying the CFIR-informed codebook (Paulus, 2023). The study team met weekly to establish a consistent understanding of the operational definitions befor and during analysis. These meetings also provided an opportunity for the lead author to review and resolve any discrepancies in coding. Upon completion of thematic coding, the team assessed code frequencies and convergence across the data to identify emerging themes within each CFIR domain, allowing for a comprehensive understanding of the factors influencing the breastfeeding environment in hospital settings.

## Results

### Description of Study Participants

The study included 12 participants, of whom 8 were lactation consultants and 4 were nurses. At the time of the study, 3 of the participants were employed at hospitals with a Baby-Friendly designation. The three hospitals that had a Baby-Friendly designation were part of the same nonprofit academic medical system. On average, these hospitals had maintained their Baby-Friendly designations for 7.3 years.

### Coding

Using our CFIR-informed codebook, we applied a code to each instance where a participant’s statement reflected the corresponding construct. The number of times a code was applied ranged from a low of 2 (Human-Equality Centeredness) to a high of 65 (Policies and Laws). Table 2 presents the frequency of code application for each code, organized by CFIR domain, along with the number of transcripts in which each code appeared. For each domain, we describe the construct that was mentioned by the greatest number of participants (i.e., the highest transcript count). When two or more constructs were mentioned by an equal number of participants, we selected the construct with the highest total frequency of code application as the tiebreaker. As such, we present the construct that emerged as most salient across participant transcripts within each of the five CFIR domains: Innovation Complexity (Innovation Domain), Policies and Laws (Outer Setting), Access to Knowledge and Information (Inner Setting), Mid-level Leaders (Individual Characteristics), and Assessing Needs (Implementation Process). The in-depth description below includes illustrative quotes to highlight key insights from participant responses. Feedback from participants collected during member checking confirmed the relevance of the identified themes, supporting the validity of our findings.

**Table 2:** Frequency of code application across CFIR domains (*n*=12)

CFIR domain	Construct	Number of codes	Number of transcripts
Innovation			
	Innovation complexity	42	12
	Innovation evidence-based	12	4
Outer setting			
	Policies and laws	65	12
	External pressure	39	11
	Local attitudes	17	7
	Critical incident	12	5
	Financing	6	4
Inner setting			
	Access to knowledge & information	59	12
	Work infrastructure	42	11
	Available resources	42	10
	Recipient-centeredness	40	12
	Materials and equipment	36	12
	Tension for change	31	10
	Physical infrastructure	28	9
	Learning-centeredness	20	7
	Relative priority	18	9
	Communications	14	7
	Funding	12	5
	Mission alignment	10	6
	Space	4	3
	Culture	4	2
	Human equality-centeredness	2	2
Individuals			
	Mid-level leaders	38	11
	Implementation leads	25	9
	Implementation facilitators	20	6
	High-level leaders	10	6
	Opinion leaders	8	5
Implementation process			
	Assessing context	56	10
	Assessing needs	48	11
	Doing	26	10
	Teaming	22	11
	Adapting	20	8

### Qualitative Findings

#### Innovation Domain: Innovation Complexity

Innovation Complexity was the most frequently coded construct within the Innovation Domain, applied 42 times across all 12 transcripts (Table 2). Participants describe breastfeeding in hospital settings as complex, with success influenced by the birth experience, such as mode of delivery [e.g., cesarean section (C-section) vs. vaginal birth], hospital policies, medical practices, cultural beliefs, and access to resources. Participants believed that most mothers admitted to maternity wards want to breastfeed as they feel it is the best option for their babies, but physical challenges such as sedation post-C-section and a lack of social and structural support often derail their plans. All 12 participants identified the high C-section rate as a barrier to breastfeeding due prolonged mother-baby separation. Participants described C-section recovery as difficult, noting that sedation, vomiting, and post-operative immobility prevent mothers from early breastfeeding initiation, within the first hour of birth. Additionally, participants expressed concern that early formula supplementation, due to this separation, can create a ‘domino effect’ that hinders exclusive breastfeeding.

One participant at a non-BFHI said,

“So, for C section, it’s a little harder for us to do skin-to-skin until the mom gets to recovery to PACU. They do hold the baby in the OR for maybe like five to 10 minutes. And then once they get to PACU, as long as they want.” – Participant 12.

While one participant at a BFHI said,

“So within, obviously, the sooner the better will be there are some moms that are all about it, you know, they’re still like up in stirrups and they want to latch the baby. Some people like to finish with being clean and comfortable, especially in the ORs, we usually don’t start the skin-to-skin process until Mom has been closed up and cleaned and moved to the stretcher. Just because it’s so cold in the OR and mom’s are under a lot of anesthesia. And a lot of the times they get nauseous, they get loopy. They don’t have a partner that with them the whole time. And it’s just a risk for the baby. It’s a risk for mom, it’s a risk for everybody. So in those cases, we usually just wrap the baby up and we’ll let the partner or we’ll hold it. And then once they’re wrapping up with the C-section, as soon as mom is put on the stretcher, we put the baby skin to skin. And most of the times in my experience, the moms latch the baby from the process of going to the OR to PA.” – Participant 1.

Participants also observed cultural and community factors that may have influenced mothers’ feelings about breastfeeding and overall breastfeeding practices, ighlighting the need for lactation consultants and staff to understand and be mindful of these influences to effectively educate patients. Participants reported that many Hispanic mothers want to breastfeed but face pressure from family members who promote formula feeding due to misconceptions about milk supply. Participants describe one common misconception among family members as the belief that ‘the baby isn’t eating enough,’ leading to unnecessary concerns about milk supply and pressure to supplement with formula. Hospital staff work to correct myths, providing visual demonstrations of the size of newborns’ stomachs to show mothers how much milk their baby actually needs. Participants also identified other common misconceptions include: “Breast milk hasn’t come in yet, so formula is needed.“; “The baby needs more than colostrum.”

Another common theme that influences the complexity of breastfeeding initiation in the hospital setting is strict medical protocols, such as those for monitoring bilirubin and glucose levels in newborns, which often prioritize formula supplementation over breastfeeding. Participants expressed that some healthcare providers fear legal liability and pressure mothers into formula feeding to prevent hospital readmissions. For example, one participant said,

“Some doctors like their blood sugar a little higher than others…if they [mom] don’t seem that motivated to continue [breastfeeding], they [doctor] might order supplementation. So that is a problem, I think that we run into, getting the moms to continue breastfeeding, because sometimes they might supplement for those reasons, you know? Or they might get a little worried if they tell them ‘oh, you gotta make sure that baby eats well, because the blood sugar will drop.’” – Participant 3.

Participants noted that some healthcare providers worry about the potential risk of NICU admission and pressure mothers to use formula feeding to avoid hospital readmissions. One lactation consultant said:

“Formula is very easy for the parents to follow up and we really need to work hard for the breastfeeding moms. Our breastfeeding rate is really low, it used to be higher. But with all the protocols and all the extra checklists of protocols that the doctors and the neonatology, [it’s]its difficult sometimes for a breastfeeding mom to continue breastfeeding. I can compare both hospitals [I worked at] one is completely going to breastfeed in newborn stomach volume, paced feeding if it is needed, bilirubin protocols, glucose protocols. And here it is completely completely, completely different. We need to supplement a lot and sometimes I had to advocate for my patients. It was difficult to not just work with the parents and give them education that they need. But at the same time, it’s very difficult to navigate through the protocols and the knowledge that the neonatologist and nurse practitioners have about breastfeeding.” – Participant 1.

Several hospitals have adopted innovative practices to address complex breastfeeding situations. Some offer donor milk for mothers with delayed lactation or preterm infants, hoping to preserve an all-human-milk diet rather than defaulting to formula. Virtual lactation services (telehealth) and phone follow-ups provide additional postpartum support; however, technology barriers and insurance/payment complexities may limit access to these services.

#### Outer Setting: Policies and Laws

Policies and Laws was the most frequently coded construct overall, applied 65 times across all 12 transcripts (Table 2). All participants acknowledged the role of policies and laws that support the initiation of breastfeeding at birth. Many noted that the BFHI influenced their hospital’s practices, regardless of whether they held the official designation. When asked what, if any, policies their hospitals had in place to support breastfeeding, providers whose hospitals did not have the BFHI designation described how their hospital incorporated several BFHI-aligned practices into their protocols and outlined their successes and challenges. For example, hospitals aim to meet BFHI standards with aligned protocols such as skin-to-skin contact, rooming-in, delayed bathing, and breastfeeding initiation within the first hour of birth. Policies also discourage the use of bottles, pacifiers, and formula unless medically indicated. In fact, the most common code co-occurrence was between Policies and Laws and External Pressure (n=16 applications), demonstrating how hospital systems may experience both regulatory and external pressure to align with the BFHI as the gold standard. As such, to maintain alignment with the BFHI, some participants discussed how formula is provided only with a doctor’s order and that its distribution is carefully controlled to prevent overfeeding. One participant shared, “We don’t just hand off the formula. There’s a process that we have to go through.” Hospital personnel educate parents on proper formula feeding when necessary. In BFHIs, nurses undergo initial training and periodic refreshers to ensure compliance with BFHI standards, including a focus on practical skills and updated policies. For example, when asked about mother-baby separation, one lactation consultant employed at a Baby-Friendly hospital said:

“It’s [mother-baby separation] minimal. Usually if they’re having a procedure like a circumcision, they’re separated and then the hearing screeners if they aren’t able to have that quiet environment, and that’s less than I mean five minutes, less than 10 minutes and Baby Friendly does allow for up to one hour-that said 23 to 24 hours the babies in the room and I can guarantee the babies are in there 99% of the time of the day yeah so yeah because we don’t have a place to put them up.” – Participant 7.

Another lactation consultant at a non-Baby-Friendly hospital described how logistical barriers prevent full compliance with Baby-Friendly standards:

“[…] there’s a little separation from that transition of labor room to mother-baby. And that’s where we fall short too because you know, it would be no separation that you know, to comply with Baby Friendly Hospital. So, there is a two to four-hour separation.” – Participant 4.

Skin-to-skin contact was one particularly common challenge when it came to C-section births versus vaginal births, and where the BFHI designation was so clearly delineated. One participant explained that for mothers with vaginal deliveries, the staff aims to maintain 60-minute skin-to-skin after birth, but for mothers with C-section deliveries, they are “lucky to get like 20 minutes in the OR.” Participants employed in Baby-Friendly hospitals discussed the requirements of sufficient staffing to guarantee immediate skin-to-skin contact following a C-section, whereas providers in non-Baby-Friendly hospitals commonly cited frustration with their inability to secure skin-to-skin under similar circumstances.

#### Inner Setting: Access to Knowledge and Information

Access to Knowledge and Information was the most frequently coded construct within the Inner Setting, applied 59 times across all 12 transcripts (Table 2). Access to knowledge and information can be broadly categorized into two key categories: training and education for hospital staff, and parental education and support. All hospitals provided some form of formal provider training initiatives at varying degrees of intensity. One participant discussed how at their hospital, new hires must complete a 20-hour lactation education course within six months of hiring, plus a five-hour hands-on skills lab. Similarly, other hospitals provide annual refresher courses, baby-friendly policy updates, and hands-on skill checks. Many nurses are encouraged to complete the Certified Breastfeeding Counselor (CBC) certification, with some hospitals reimbursing the cost.

One lactation consultant reflected on their hospital’s education initiatives, saying:

“We’re really working towards getting all of our nurses so we’re talking postpartum, L&D and NICU and nursery nurses. In fact, like all of them, we were training them to do the CBC course. So, we’ve had 74 nurses go through it already.” – Participant 7.

Hospitals also actively train physicians in breastfeeding knowledge, to promote physician-led education to ensure patients receive breastfeeding support. For example, one participant noted that their hospital adopted “train the trainer to train the hospital staff in lactation competencies.” Some hospitals distribute educational binders to community physicians, providing trimester-based breastfeeding education. However, budget constraints and supply chain issues affect access to educational materials (e.g., breastfeeding books are on backorder). One participant detailed the robust physician training process at their hospital:

“So, we are not we don’t have an affiliate clinic, per se, but we have community physician that bring their patients here. So, we educate our providers, they had to all take an online class, provide us with their certificates showing that they’ve completed the material. And then we provide them with a binder. And in that binder, it has broken down different flyers and educational topics that should be covered. Now, whether they cover it, I don’t know or what they you know, they’re free to use their own information. But this is just something that we have done as part of our community outreach that we are, you know, providing something from the hospital. And then of course, our director, she also does site visits, just to touch base with our providers that the services we provide, and you know, hopefully, they’ll bring their patients to us.” – Participant 7.

As for parental education and support, some hospitals lack dedicated lactation consultants for night shifts, leading to gaps in support when mothers are most likely to struggle, and nurses are often overburdened, which makes it difficult to provide thorough breastfeeding assistance. Free prenatal, breastfeeding, and childbirth classes are offered at some hospitals, in both English and Spanish. Classes include topics such as newborn feeding cues, managing engorgement, and preparing for breastfeeding success.

“We also have one of our childbirth educators who hosts a new mom group, like a support group for moms after they deliver. If the moms are having any issues with breastfeeding, then she gives them our number in order for her to call. If it’s something that we can discuss over the phone, you know, an issue of her milk supply, how can she increase it? You know, she’s sore, how can she treat it? We speak to her or whatever questions you may have. If it’s an issue where we should actually see her feed then through the [Hospital System] Care on Demand app, there are actually breastfeeding counselors and lactation consultants that she can actually make an appointment with, to Skype and actually help her with a feeding.” – Participant 10.

However, staff turnover influences the ability of hospitals to deliver sufficient knowledge and information to patients, as new nurses often lack experience or proper training. Similarly, limited staff may reduce the capacity of existing efforts to reach as many patients as possible with direct breastfeeding support services. Many hospitals have only one lactation consultant or a very small team serving all postpartum patients. Some hospitals provide weekend or evening lactation consultant coverage, but in others, no one is available outside of weekday business hours. This often leaves staff nurses, who are already busy, to handle breastfeeding issues at night or on weekends, making educating patients even more difficult.

Cultural and community influences contributed not only to the complexity of breastfeeding, but also to the ways in which mothers interacted with access and knowledge provided in the hospital setting. Participants discussed how some families prioritize formula feeding due to generational beliefs, concerns about milk supply, or the perception that mixed feeding (‘los dos’ or ‘both’) is best. Many participants expressed that there is not enough education before mothers make it to the hospitals, so lactation consultants feel like they are battling for families to understand the importance of breastfeeding. Participants discussed prenatal education more frequently than postpartum education, potentially due to the fact that participants felt mothers had already decided their infant feeding practices prior to labor and delivery. One participant highlighted that, “If the OBGYNs provide the necessary education about breastfeeding, and the nurses do too, and the neonatologists are on the same page, it’s going to be easier to successfully help breastfeeding.” Another participant suggested “OB education” as a feasible strategy for improving breastfeeding within the hospital. These sentiments point to both the need for earlier breastfeeding education and cohesive messaging from prior to conception through delivery.

In addition to providing breastfeeding support during labor and delivery, hospitals implement strategies to provide families with essential educational resources at discharge to help them care for their newborns. Hospitals provide discharge booklets covering postpartum care, breastfeeding guidelines, and newborn care. Nurses review feeding expectations, diaper output, and signs of effective breastfeeding prior to discharge. At some hospitals, lactation consultants are available for outpatient consultations, phone support, and virtual assistance via telehealth platforms. Hospitals often partner with WIC, La Leche League, and Healthy Start to provide additional breastfeeding resources. Notably, two participants referenced the use of QR codes on discharge materials that direct patients to breastfeeding video tutorials and a chart on milk storage.

#### Individuals Domain: Mid-level Leaders

Mid-level Leaders was the most frequently coded construct within the Individuals Domain, applied 38 times across 11 of 12 transcripts (Table 2). Lactation consultants, as the implementation leads, expressed frustration with the lack of coordination and support from various healthcare providers and administrators. While some professionals advocate for exclusive breastfeeding, many prioritize medical convenience, legal concerns, or institutional policies over evidence-based lactation support. This disconnect between different roles in the hospital leads to inconsistent breastfeeding guidance, creating barriers for new mothers trying to exclusively breastfeed.

Physicians, as mid-level leaders, have a great opportunity for breastfeeding influence; however, their different responsibilities often are prioritized over breastfeeding. For example, many obstetricians do not prioritize breastfeeding education during prenatal care, missing opportunities to prepare mothers before delivery. Neonatologists often prioritize medical stability over breastfeeding, leading to routine formula supplementation for issues like low blood sugar, jaundice, or prematurity. Neonatologists sometimes order supplementation before lactation consultants can intervene, bypassing efforts to establish breastfeeding. One participant suggested the need for physician-specific education to foster a more unified and effective effort, expressing that, “Doctors listen best to doctors. So maybe having a physician group or physician entity like Academy of Breastfeeding Medicine or Academy of Physicians address physician to physician about the importance of this [breastfeeding].” Further, many lactation consultants believed that community pediatricians set unrealistic child weight gain expectations, leading mothers to think their baby isn’t getting enough milk. The lack of standardized guidance between hospital-based and community-based pediatricians leads to confusion for mothers.

Hospital management, like nurse leaders and nurse directors, has significant influence over breastfeeding policies, but support varies by institution. Some nurse directors prioritize formula feeding for convenience, particularly when staffing shortages make it difficult to provide breastfeeding support. Some nurse directors approve additional lactation support measures, such as outpatient consults for struggling mothers, but these programs are not always well-funded or widely implemented.

Finally, lactation consultants shared concerns that administrators’ focus on financial and operational priorities often leads to breastfeeding initiatives being deprioritized. A lack of investment in lactation consultant staffing, prenatal education, and ongoing staff training can undermine the effectiveness of breastfeeding efforts. Furthermore, renovations and facility constraints, driven by budget limitations, sometimes delay the implementation of Baby-Friendly Hospital initiatives, such as ensuring mothers and babies stay together after C-sections. The COVID-19 pandemic exacerbated breastfeeding support challenges, with some hospitals temporarily halting WIC peer counselor programs and in-person prenatal education. Additionally, some hospitals offer donor milk as an alternative or supplement to formula, but the provision of donor milk relies heavily on administrative buy-in. These competing priorities highlight how operational decisions directly influence the ability of the hospital team to provide comprehensive breastfeeding support.

#### Implementation Process: Assessing Needs

Assessing Needs was the most frequently coded construct within the Implementation Process domain, applied 48 times across 11 of 12 transcripts (Table 2). Lactation consultants assess the specific needs of mothers and staff to provide tailored breastfeeding support. They often have limited contact hours to train staff or sit with mothers, despite anesthesia effects (which are particularly relevant for C-section deliveries), and emotional readiness, to ensure proper breastfeeding support. One-on-one assessments enable consultants to identify specific challenges such as poor latch, sore nipples, or timing concerns (like a sleepy newborn). While hospitals promote exclusive breastfeeding, consultants respect mothers’ feeding choices (breastfeeding, combination feeding, or formula). They assess families’ concerns whether it’s pain, fear of low supply, or cultural beliefs, and tailor guidance on safe formula usage or how to maintain breast care if not nursing. The goal is to avoid pressuring mothers while still offering evidence-based insights on how supplementation affects supply, the baby’s stomach capacity, and long-term feeding patterns. They try to spend as much of their time educating their patients as they do learning about them:

“When I’m there I go into every single room, regardless of her feeding choice or not. Because I think I like to sit down in her room and talk with her and listen to her story. You’d be surprised how many people didn’t know the benefits of breastfeeding, you know, or my nurses, sometimes they have 10 patients, and they don’t get to talk that long to the mom about breastfeeding, they’ll just do like, whatever question she asked and then that’s it. You know, so I tried to sit down and fill the holes, to really, you know, try to inspire to try it out, you know.” – Participant 4.

They tailor approaches for different populations. Babies in NICU, late preterm/small for gestational age infants, or those with borderline blood sugars often require extra monitoring and potential supplementation. Lactation staff assess each baby’s feeding readiness and tailor their approach (e.g., syringe feeding, hand expression) before resorting to formula. One lactation consultant discussed how many migrant mothers prefer breastfeeding but struggle with work constraints and dialect-language barriers make education challenging, requiring more visual and hands-on instruction. Some mothers supplement due to past traumatic breastfeeding experiences. Lactation consultants use a compassionate approach, educating without judgment and offering non-pressured support. Strategies like syringe feeding, paced bottle feeding, and minimizing pacifier use, and hand expression help preserve breastfeeding potential.

## Discussion

Miami-Dade County hospitals generally demonstrated a strong commitment to Baby-Friendly principles and evidence-based breastfeeding practices, however, several implementation barriers limited consistent delivery of breastfeeding support services. High cesarean rates, limited lactation consultant staffing (especially on nights and weekends), and conflicting neonatal protocols challenge hospitals’ efforts to achieve full BFHI compliance. Nonetheless, participants continuously adapt to provide breastfeeding and education services within their unique hospital settings. Furthermore, through these interviews, and when possible, in their work, participants advocate for improved policies, resource allocation, and educational initiatives to address the diverse needs and contexts of the families they serve. By promoting collaboration across departments, offering robust prenatal and postnatal education, and honoring each mother’s autonomy, many institutions are working toward more equitable, accessible, and supportive breastfeeding environments.

### Innovation Domain: Innovation Complexity

C-section deliveries consistently emerged as a major barrier to early breastfeeding initiation. Florida ranks among the states with the highest C-section rates in the country at 35.9% (National Center for Health Statistics, 2022). Given that this study was conducted in Florida, we can infer that the state’s high C-section rates, including 70% in one hospital, contributed to routine maternal-infant separation and delayed initiation of breastfeeding. These practices directly contradict Step 4 of the BFHI, which encourages skin-to-skin contact and early initiation of breastfeeding within one hour of birth (World Health Organization, 2009). Participants noted that many mothers are sedated or in pain post-operatively, complicating efforts to latch and breastfeed. Even in hospitals that attempted to implement skin-to-skin contact in the operating room, logistical and staffing limitations often increased the complexity of breastfeeding initiation and curtailed its feasibility. These findings mirror the broader literature demonstrating that C-section births are associated with lower breastfeeding initiation and duration (Li et al., 2021). A recent literature review reported on the perceptions and experiences of breastfeeding among mothers who had C-sections, finding that key challenges include physical limitations after surgery, wound pain, stress, fear, and breastfeeding failure (Li et al., 2021). One study evaluated the efficacy of an intervention on women with planned C-sections to establish positive breastfeeding attitudes, cultivate supportive subjective norms, enhance perceived behavioral control, and strengthen breastfeeding intention to change behaviors (Wen et al., 2021). This intervention included prenatal education, familial support, massage therapy, and post-discharge support, and found that the intervention group had higher exclusive breastfeeding rates than the control group at 5 days, 2 weeks, and 1 month. Importantly, the intervention was intentionally designed to address known barriers to breastfeeding after C-section and demonstrated that when women receive appropriate education, social support, and responsive care, they are more likely to initiate and sustain exclusive breastfeeding.

### Inner Setting: Access to Knowledge and Information

Prenatal education also emerged as a critical factor in establishing breastfeeding intentions and confidence. Participants consistently emphasized that many mothers arrived at the hospital with limited or outdated knowledge, often shaped by cultural norms or family influence. Yet, participants observed that OB/GYNs rarely incorporated breastfeeding education into routine prenatal care, missing opportunities to prepare mothers before labor and delivery. This gap was especially concerning for first-time (Massare et al., 2023) or high-risk mothers (Salama et al., 2024), who would benefit most from anticipatory guidance. Most women make their decision to breastfeed in preconception or early pregnancy and studies have found that a physician’s explicit recommendation to breastfeed initiation, continuation, and exclusivity rates have a significant influence on their decision (Mass, 2015). A major barrier to breastfeeding education for physicians is that many feel unprepared to discuss breastfeeding with patients (Esselmont et al., 2018; Freed et al., 1995; Krogstrand & Parr, 2005), as reflected in national data showing significant knowledge gaps, limited clinical training during residency, and a widespread perception that breastfeeding education in medical training is inadequate. Regardless, mothers who believe their provider encourages breastfeeding are significantly more likely to initiate breastfeeding than those who did not receive encouragement (Lu et al., 2001). Additionally, prenatal breastfeeding education increases women’s knowledge of breastfeeding which in turn has a positive effect on breastfeeding initiation and exclusivity (Kehinde et al., 2023). Many of the participants in this study described the prenatal classes that their hospitals offered. These classes are a potential point of intervention where OB/GYNs could initiate breastfeeding education and demonstrate breastfeeding support, providing patients with a trusted source of information early on and addressing misconceptions while mothers are planning their infant feeding strategy.

### Individuals Domain: Mid-level Leaders

Lactation support staffing shortages, especially on nights and weekends, were a pervasive concern. Some hospitals had only one or two lactation consultants covering dozens of postpartum beds, limiting their ability to conduct thorough assessments, providing timely education, or following up with struggling mothers. Although a key step of the BFHI is providing staff and physician training in basic breastfeeding management, it does not set any parameters for staffing of clinical lactation care professionals, that is, International Board-Certified Lactation Consultants (IBCLCs) (Mannel, 2025). However, the International Lactation Consultant Association has published guidelines for developing and managing a hospital lactation service that outline factors such as dedicating full-time equivalents (FTEs) for lactation consultants to ensure access to care in a timely fashion, documentation, data collection, and staff education (Mannel & Mannel, 2006; Mannel et al., 2012). Additionally, the participants of this study also identified that staff turnover and a lack of breastfeeding training among new nurses created further obstacles to high-quality care. Even in Baby-Friendly hospitals, sustaining BFHI practices depends heavily on institutional investment in staffing, space, and training. Participants emphasized that when nurse directors or administrators prioritized breastfeeding support, it improved access to resources like donor milk, outpatient consultations, and continuing education. Conversely, when hospital leadership prioritized operational efficiency or budget constraints, breastfeeding support efforts were often deprioritized. Many of these barriers and facilitators are in line with those found in other studies. A scoping review of the BFHI, revealed several key patterns: (i) strong national leadership, evidenced through legislation, funding, and policy, is essential for advancing breastfeeding; (ii) supportive hospital policies, particularly in Baby-Friendly and neonatal care settings, play a critical role; (iii) successful implementation depends on adherence to specific, evidence-based steps; (iv) the Baby-Friendly Community Initiative (BFCI) remains limited to a few countries, highlighting the need for greater government investment to scale its reach; (v) ongoing training and strengthened breastfeeding knowledge among health workers are necessary to support long-term practice changes; and (vi) educational programs targeting pregnant and postpartum women are vital to sustaining exclusive breastfeeding (Walsh et al., 2023). Together, these findings underscore that a multi-level approach, spanning policy, health systems, clinical practice, and community education, is essential to creating environments that protect, promote, and support breastfeeding, particularly in settings where structural and social barriers remain pervasive.

After the analysis of this study, the authors presented the results to our local partner, BreastfeedMIAMI, at our monthly community meeting. Community members worked together to create recommendations based on these findings. Recommendations from BreastfeedMIAMI highlight several actionable strategies to improve breastfeeding support in maternity settings. First, strengthening staff training and ensuring consistency is essential; this includes standardized breastfeeding education for all maternity staff, annual competencies, and systems to mitigate the challenges of high turnover and traveling nurses. Second, enhancing coverage and resources, such as expanding lactation consultant availability to 24/7 and ensuring timely access to electric pumps and donor milk, can significantly support milk supply and breastfeeding success. Third, aligning neonatal, obstetric, and pediatric teams on “Baby-Friendly” practices and securing leadership advocacy are critical to reducing mother–baby separation and reinforcing evidence-based care. Fourth, fostering collaborative, patient-centered care requires physician-to-physician communication, culturally responsive education, and respect for maternal autonomy. Fifth, sustained breastfeeding outcomes can be supported through ongoing education and follow-up, including prenatal classes, discharge materials in multiple languages, and accessible post-discharge support. Finally, targeted efforts are needed to address C-section-specific barriers, such as delays in skin-to-skin contact and challenges related to sedation or NICU admission. These multifaceted recommendations underscore the importance of coordinated, patient-informed care to create breastfeeding-friendly environments across the continuum of maternity care.

While this study provides valuable insights, four limitations should be considered when interpreting the findings. First, as is typical of qualitative studies, the sample size for this study is small, and participants’ experiences are specific to the contexts of maternity wards in Miami-Dade County. Second, the hospitals in Miami-Dade County serve a diverse range of racial and ethnic groups, including a majority Hispanic population, which may influence breastfeeding practices and the applicability of findings to other regions. Third, given that the sampling method for this study involved the community partner and the research team working jointly to identify individuals with in-depth knowledge of the breastfeeding climate within their hospitals, there is potential for selection bias, as perspectives from other hospital personnel, such as administrators, doctors, and physician assistants, may not have been captured. Despite this limitation, we believe that the individuals who did participate were best positioned to speak to the breastfeeding environment in their hospitals due to their daily interaction with breastfeeding mothers in labor and delivery. Lastly, due to the small size of maternity ward staff in select hospitals, the authors did not provide demographic characteristics of participants in an effort to protect their anonymity. The majority of participants in this study were lactation consultants, many of whom are the only lactation consultants in their respective hospitals. To further ensure confidentiality, the authors also withheld the leadership roles of participants and the names of the hospitals where participants were employed. Although these measures may obscure the context of each maternity ward setting, they were taken to ensure that participants could share their experiences and thoughts openly without concern of identification, ultimately strengthening the authenticity and trustworthiness of the findings.

## Conclusion

We identified multiple institutional barriers and facilitators that shape the breastfeeding environment across Miami-Dade County maternity wards. Emerging themes spanned the innovation domain, inner setting, outer setting, individual domain, and implementation process, highlighting the multifaceted determinants of breastfeeding environment in Miami-Dade County maternity wards. Participants described C-section delivery, cultural and community influences, and medical practices as major sources of complexity. Hospital policies aligned with BFHI appeared to support breastfeeding practices in both designated and non-designated hospitals, although staffing, resources, and logistical constraints limited consistent implementation. Hospitals also made a concerted effort to improve breastfeeding outcomes by increasing access to knowledge and information through staff training and parental education. Participants identified physicians, nurse managers, and administrators as critical leaders whose engagement and institutional support could strengthen breastfeeding practices. Competing priorities among these leaders may hinder consistent and effective breastfeeding support. Greater coordination, physician engagement, and institutional investment are critical to improving outcomes. Lactation consultants drive much of the implementation process by tailoring breastfeeding support to individual mothers’ needs. Targeted practice-level interventions that address known post-cesarean barriers, alongside policy-level recommendations from community partners like BreastfeedMIAMI (e.g., enhanced staff training, expanded lactation coverage, and interdepartmental alignment), can help foster more equitable, breastfeeding-friendly environments. Sustained change will require both institutional investment and compassionate, coordinated care that meets families where they are and supports their feeding goals from birth onward.

## Data Availability

Data can be available upon request.

## Appendix

Semi-Structured Interview Guide.

Key informants were asked the following grand tour questions, each developed to elicit participant experiences relevant to one or more of Baby-Friendly USA’s Ten Steps to Successful Breastfeeding (USA, 2012), and mapped to the anticipated CFIR domain and construct used in subsequent coding.

**Table:**

#	Grand Tour Question	Corresponding Ten Step(s)	Anticipated CFIR Domain (Construct)
1	How is your hospital environment or layout designed to support breastfeeding?	Step 4/Step 6	Inner Setting (Physical Infrastructure)
2	Who is available in your hospital to provide direct breastfeeding support or counseling to mothers?	Step 1c (staff competence)	Inner Setting (Available Resources)/Inner Setting (Work Infrastructure)/Individual Characteristics (Implementation Leads, Implementation Facilitators)
3	What breastfeeding resources do you have that aim to educate mothers?	Step 2 (education)	Inner Setting (Access to Knowledge and Information)
4	How does your hospital support mothers who are expressing milk?	Step 5 (separation support)	Inner Setting (Available Resources)
5	Does your hospital provide pumps and what types? If not, does the hospital assist in obtaining pumps?	Step 5	Inner Setting (Materials and Equipment)
6	Do you offer any opportunity for parents to room in with their infant?	Step 6 (rooming-in)	Outer Setting (Policies and Laws)/Inner Setting (Physical Infrastructure)
7	What written policies do you have on breastfeeding, breastfeeding-related topics, or skin-to-skin?	Step 1b (written policy)	Outer Setting (Policies and Laws)
8	What is the role of breastfeeding in discharge planning? Do you provide referrals to community resources?	Step 9 (discharge/ongoing support)	Implementation Process (Adapting)/Outer Setting (External Pressure)
9	Are there any other breastfeeding-related supports you’d like to add?	Open-ended	Captures inductive content across domains

Ten Step and CFIR domain correspondences reflect the study team’s a priori design logic linking interview content to the Baby-Friendly Ten Steps and the CFIR codebook (Table 1). Some domains (e.g., Innovation, Individual Characteristics beyond Question 2) emerged more inductively across multiple questions rather than being anticipated by a single item.

## Code Availability

Dedoose application can be available upon request.
